# Buprenorphine discontinuation and utilization of psychosocial services: a national study in the Veterans Health Administration

**DOI:** 10.1186/s13722-025-00562-1

**Published:** 2025-04-16

**Authors:** Emma N. Cleary, Angela L. Rollins, Alan B. McGuire, Laura J. Myers, Patrick D. Quinn

**Affiliations:** 1 Department of Psychological and Brain Sciences, Indiana University, 1101 E. Tenth St, Bloomington, IN 47405 USA; 2 VA HSR&D Center for Health Information and Communication, Richard L. Roudebush VA Medical Center, 1481 W. 10 th St., Indianapolis, IN 46202 USA; 3 Regenstrief Institute, Inc., Indianapolis, USA; 4 ACT Center of Indiana, Psychology Department, IUPUI, Indianapolis, IN USA; 5 Psychology Department, IUPUI, Indianapolis, IN USA; 6 Department of Medicine, Indiana University School of Medicine, Indianapolis, USA; 7 Department of Applied Health Science, School of Public Health, Indiana University, 809 E. 9 th St., 203, Bloomington, IN USA

**Keywords:** Medications for opioid use disorder, Treatment retention, Psychosocial intervention, Substance use disorder, Pharmacoepidemiology, Psychotherapy

## Abstract

**Background:**

Longer duration of treatment with medication for opioid use disorder (MOUD) is associated with improved outcomes, but long-term retention remains a challenge. Research is needed to identify psychosocial interventions that support MOUD retention. To address this gap, we examined associations between a wide range of psychosocial services and buprenorphine treatment discontinuation across 18 months among a large cohort of Veterans initiating buprenorphine nationwide.

**Methods:**

We identified a cohort of patients with new buprenorphine initiation in 2017–2018 in Veterans Health Administration electronic health record data (N = 11,704). We examined prescription fills for up to 18 months after initiation. The primary outcome was first discontinuation of buprenorphine. We examined a variety of services, including psychotherapy in specialty substance use disorder (SUD) and mental health clinics, other healthcare services, and residential programs. To examine time-varying associations between psychosocial services and risk of discontinuation, we fit extended Cox regression models for each service separately and simultaneously.

**Results:**

Overall, 80.5% of patients discontinued buprenorphine at least once within 18 months. Risk of discontinuation was 18% (HR: 0.82, 95% CI: 0.77, 0.87) relatively lower following SUD psychotherapy and 26% (HR: 1.26, 95% CI: 1.15,1.39) higher following residential treatment.

**Conclusions:**

Several services, including residential treatment, were associated with greater risk of subsequent buprenorphine discontinuation, whereas only SUD psychotherapy was consistently associated with lower risk of later discontinuation. These findings emphasize the need for future studies to increase understandings of beneficial and disruptive components of psychosocial services to improve treatment retention among patients receiving MOUD.

**Supplementary Information:**

The online version contains supplementary material available at 10.1186/s13722-025-00562-1.

## Introduction

Opioid use disorder (OUD) is a major public health crisis in the United States [1], and opioid overdose deaths among Veterans continue to rise substantially [2–4]. Veterans are considered to be a group at high risk of OUD due to their higher prevalence of risk factors like chronic pain and psychiatric comorbidities compared with the general population [5–7]. The US Department of Veterans Affairs (VA), Veterans Health Administration (VHA) is the largest direct provider of substance use disorder (SUD) treatment (medication and/or psychotherapy) in the United States [8]. Medications for opioid use disorder (MOUD) include buprenorphine, methadone, and naltrexone and are considered the criterion standard of treatment for OUD [9–11]. There is strong evidence that buprenorphine and methadone, especially, are effective in reducing illicit opioid use [11–13] and risk of overdose [14, 15].

Duration of continuous MOUD pharmacotherapy may be an important clinical process indicator, given that gaps in buprenorphine treatment greater than 2 weeks have been associated with increased overdose risk [16]. Mortality rates and other clinical outcomes, like longer periods of continuous abstinence, improve with longer MOUD treatment duration [14, 17, 18]. A minimum of 180 days of continuous pharmacotherapy has been recommended, although this duration may be insufficient for many patients [19–21]. However, long-term MOUD retention remains a challenge, as the average length of time in treatment is often less than 6 months [22, 23]. Challenges with long-term retention have also been demonstrated among Veterans, despite efforts by the VA to expand access to MOUD treatment [24–27]. Existing research indicates that sociodemographic and clinical characteristics such as Black racial identity, younger age, and frequent emergency room visits are associated with shorter buprenorphine course duration [24] and greater risk of discontinuation among Veterans [25].

The VHA offers a variety of psychosocial and other services for Veterans, including health care services like primary care and pain management, mental health services including treatment for post-traumatic stress disorder (PTSD), and rehabilitation programs that range from outpatient recreational therapy and vocational services to residential programs for Veterans experiencing homelessness. In general, few adjunctive interventions have been identified that have the potential to improve MOUD retention [28–31], and little is known about the potential benefit (or harm) of engagement with specific types of VHA psychosocial services with respect to MOUD treatment retention, with the exception of one study of trauma treatment in a single VHA buprenorphine clinic [6]. There is less research on the impact of other VHA psychosocial interventions on MOUD treatment retention, especially regarding continuous treatment and retention beyond 1 year, as the few previous studies on long-term treatment retention in Veterans have not accounted for gaps in treatment [24, 25, 30]. Thus, there is a need to explore, on the one hand, whether VHA services can help patients stay in treatment or, on the other hand, whether involvement with certain services may make MOUD retention more difficult or even discourage retention. As one example of the latter, some abstinence-based residential recovery programs may discourage admission of residents on MOUD, and other programs may admit these patients but encourage tapering MOUD while enrolled [32, 33].

The current study examined buprenorphine treatment retention across 18 months using longitudinal VHA records. We first aimed to describe long-term retention among a large cohort of patients initiating buprenorphine nationwide. We focused on sublingual buprenorphine and buprenorphine-naloxone combinations given that methadone treatment is largely outsourced to community care and research suggests that buprenorphine may be superior to extended-release naltrexone (e.g., due to induction failure) [8, 34]. Given documented disparities in retention in MOUD treatment among racial/ethnic minorities, we also aimed to examine whether discontinuation rates vary based on sociodemographic characteristics. The third and most critical aim of this study was to examine associations between a diverse array of psychosocial and medical services and risk of buprenorphine treatment discontinuation, with the goal of identifying potential areas of intervention for patients receiving MOUD. To our knowledge, this is the first study to examine a wide range of VHA psychosocial services, from mental health and substance use treatment to medical and rehabilitation services, and their relationships with continuous, long-term buprenorphine retention.

## Methods

### Cohort and data source

In this cohort study, we identified Veterans with new buprenorphine initiation in 2017–2018 using electronic health record data extracted from the VHA Corporate Data Warehouse (CDW). CDW is a national repository of clinical and administrative information that is housed at the VA Informatics and Computing Infrastructure (VINCI) and is obtained from the VA electronic medical record system. CDW data included prescription records, inpatient and outpatient files, healthcare utilization, and sociodemographic information. The index date was defined as the first new buprenorphine fill date between January 1 st, 2017, and December 31 st, 2018, without a history of buprenorphine prescription in the 6 months (180 days) prior to the index date. For patients with multiple eligible buprenorphine initiation dates, we selected the first initiation date, such that each patient was included in the analysis only once. To capture buprenorphine medications for OUD treatment only, we excluded prescriptions with formulations (i.e., intravenous, transdermal patch, and buccal film) used in pain treatment [35]. The vast majority of patients in our primary analysis cohort had engaged with psychotherapy for SUD at some point during the study period (88.1%) while only a quarter of patients (26.0%) were involved in pain treatment, increasing confidence that the included buprenorphine prescriptions were for treatment of OUD rather than pain.

We examined prescription fills for up to 18 months after the index date or until death or end of study, which we defined as March 1 st, 2020 to account for changes in buprenorphine delivery as a result of the COVID- 19 pandemic [36], meaning that some individuals were followed for less than 18 months by design. To examine changes in service use over time, follow-up time was partitioned into six 90-day quarters. Analyses were performed using SAS Enterprise Guide version 8.3 (SAS Institute Inc., Cary, NC) from April 2023 through November 2024.

### Outcome

The primary outcome was first discontinuation of buprenorphine treatment. We defined discontinuation as the first quarter in which an individual had 76 or fewer days of buprenorphine (i.e., at least 14 days without buprenorphine in a quarter, continuously or cumulatively), identified through records of dispensed prescriptions. This definition was informed by previous MOUD retention definitions and findings that gaps in treatment greater than 2 weeks are associated with increased risk of overdose [16, 26, 27, 37]. Given the person-quarter data structure, the 14-day criterion in this study did not require gaps to be contiguous. Sensitivity analyses, described below, additionally examined the robustness of our results to a less strict definition.

### Services

The primary aim of this study was to examine associations between engagement with a range of psychosocial and other services and risk of buprenorphine treatment discontinuation. We included services aimed at treating mental health and SUD symptoms, which may impact MOUD retention [38]. These included SUD psychotherapy, largely provided within specialty SUD clinics, as well as individual and group services provided within specialty mental health clinics. We also captured brief mental health treatment delivered by primary-care-based mental health providers. We included PTSD treatment, as symptoms may undermine effectiveness of buprenorphine treatment [6]. Intensive residential treatment program services, often designed for Veterans with mental health disorders, SUD, and/or experiencing homelessness, were also included. All services were identified using VA-specific service codes called stop codes.

Medical burden is associated with MOUD discontinuation among Veterans [25], so we included services related to management of medical comorbidities: primary care, pain services, and clinical pharmacy. Specialty pain providers help to manage pain with medications and non-pharmacological modalities, and clinical pharmacists provide direct patient care via comprehensive medication management. Finally, we included a range of services aimed at improving social and occupational functioning and quality of life: social work, chaplain services, vocational services, criminal justice outreach, and recreational or occupational therapy. Social workers within the VA perform clinical interventions and programming including outreach, counseling, and case management. VA chaplains provide religious and spiritual care. Vocational services provide job training and education, as well as vocational rehabilitation based on the individual place and support model [39]. Criminal justice outreach services facilitate access to criminal diversion and therapeutic court programs for justice-involved Veterans [40]. We included recreational and occupational therapy intended to maintain or improve functional independence and life quality. All these services provide resources that could potentially assist with overcoming barriers to MOUD treatment retention.

We created dichotomous indicators for each service that assessed any use for each quarter, such that they could change in value for each quarter over the course of follow-up. To ensure temporal precedence (i.e., that service receipt preceded discontinuation), we lagged time-varying covariates and service predictors by one quarter in all analyses. That is, individuals were considered exposed in a quarter if they were involved in the service in the prior quarter. For the first quarter of follow-up, individuals were considered exposed if they were engaged with the service in the quarter prior to buprenorphine initiation. Lagging these covariates also accounted for the possibility that engagement with a service may not have an immediate effect on treatment discontinuation.

### Covariates

We included time-stable sociodemographic covariates for gender, race [41], and age at first buprenorphine initiation. We also included two time-varying covariates. The first was a lagged indicator of any emergency department (ED) visits, which we included to reduce confounding based on previous research that identified associations between ED visits and buprenorphine discontinuation [25]. The second was a lagged indicator of receipt of buprenorphine from multiple unique facilities, which may reflect inappropriate buprenorphine treatment, misuse, or barriers to treatment such as pharmacy stock issues or provider error [42].

### Statistical analyses

We began by calculating cumulative risk of discontinuation, using the Kaplan–Meier method to account for right-censoring by March 1 st, 2020, or death. To evaluate whether those who utilized each service were more or less likely to discontinue buprenorphine after receiving the service relative to those not utilizing the service, we fit extended Cox regression models with time-stable and time-varying covariates, adjusted for clustering by initial buprenorphine facility. We first assessed whether each service was associated with risk of buprenorphine discontinuation in separate models for each service. These models were minimally adjusted for sociodemographic characteristics, ED visits, and receipt of buprenorphine from multiple facilities. Next, to account for the possibility that Veterans were engaged in multiple services at the same time, we fit one fully adjusted model. This model included all services simultaneously to assess whether associations remained for any services when holding constant the other services, as well as the other covariates. We used the false discovery rate *P*-value correction for multiple comparisons in the fully adjusted model only.

We performed three sensitivity analyses to examine the robustness of results from the fully adjusted model. As many Veterans discontinue MOUD less than a year after initiation [24–27], we examined associations with discontinuation by 6 months. We also repeated analyses with a less-restrictive discontinuation definition of 30 days without buprenorphine, which recent research supports as a valid definition for treatment discontinuation [21]. Finally, we assessed buprenorphine receipt via outpatient prescription records, meaning that we could not capture receipt of buprenorphine via other sources. To avoid immeasurable time bias, we examined associations after excluding follow-up quarters in which individuals spent at least 14 days in an inpatient or residential setting [43]. These individuals could re-enter follow-up in subsequent quarters that had fewer inpatient days.

## Results

### Cohort characteristics

We initially identified a cohort of 11,770 Veterans with new buprenorphine initiation. After excluding individuals who died during the first follow-up quarter (n = 66; Figure S1), the final study cohort consisted of 11,704 Veterans (99.4% of the initial cohort), most of whom were male (91.4%), and white (81.2%, Table 1). The most common age category was 30–39 (32.0%). The median follow-up until discontinuation or censoring was 1 quarter [interquartile range (IQR):1–4)].

**Table 1:** Demographic characteristics of patients with new buprenorphine initiation

Characteristic	N	%
Age		
20–29	1109	9.5
30–39	3743	32.0
40–49	1582	13.5
50–59	2271	19.4
60–88	2999	25.6
Gender		
Female	1002	8.6
Male	10,702	91.4
Race		
Asian	61	0.5
Black	1570	13.4
Other	164	1.4
Unknown	400	3.4
White	9509	81.2

The “other” race/ethnicity category includes Hispanic or Latino, Native Hawaiian or other Pacific Islander, American Indian or Alaskan Native, or other races and ethnicities. In some cases, information on race/ethnicity was missing and was categorized as “unknown”

Kaplan–Meier estimates of buprenorphine discontinuation indicated that, across all demographic categories, more than 60% of patients in this study had discontinued buprenorphine at least once within 6 months of initiation, and more than 80% discontinued within 18 months (Table 2). Absolute risk of treatment discontinuation within 6 months was highest among individuals aged 20–29 (71.6%). Rates of discontinuation increased to 86.2% within 18 months for this group. Regarding race/ethnicity, risk of 6-month (71.1%) and 18-month discontinuation (86.9%) was highest among Black individuals and lowest among Asian individuals (62.3% and 73.9%, respectively). Rates of 6-month discontinuation were slightly higher among female Veterans (65.8%) relative to male Veterans (63.4%), although 18-month rates were similar for both groups (female: 81.2%; male: 80.5%).

**Table 2:** Kaplan–Meier estimates of cumulative buprenorphine discontinuation overall and stratified by demographic characteristics

Characteristic	6 months (95% CI)	18 months (95% CI)
Overall	63.6% (62.7%, 64.5%)	80.5% (79.8%, 81.3%)
Age		
20–29	71.6% (69.0%, 74.3%)	86.2% (84.2%, 88.3%)
30–39	65.5% (63.9%, 67.0%)	81.2% (79.9%, 82.4%)
40–49	63.5% (61.2%, 65.9%)	79.6% (77.5%, 81.6%)
50–59	59.7% (57.7%, 61.7%)	79.1% (77.4%, 80.8%)
60–88	61.4% (59.6%, 63.1%)	79.2% (77.8%, 80.7%)
Gender		
Female	65.8% (62.8%, 68.7%)	81.2% (78.8%, 83.7%)
Male	63.4% (62.5%, 64.3%)	80.5% (79.7%, 81.2%)
Race		
Asian	62.3% (50.1%, 74.5%)	73.9% (62.8%, 84.9%)
Black	71.1% (68.8%, 73.3%)	86.9% (85.1%, 88.6%)
Other	64.6% (57.3%, 72.0%)	83.9% (78.1%, 89.8%)
Unknown	66.8% (62.1%, 71.4%)	83.0% (79.2%, 86.7%)
White	62.2% (61.3%, 63.2%)	79.4% (78.5%, 80.2%)

95% CI = 95% Confidence Interval

### Services as predictors of MOUD discontinuation

The mostly commonly used services were primary care, mental health clinic services, and SUD psychotherapy, while the least commonly used services were criminal justice outreach, vocational services, and post-traumatic stress disorder treatment (Table S1). In separate Cox regression models for each service (adjusting for age, gender, race, ED visits, and receipt of buprenorphine from multiple facilities), several services were associated with increased risk of discontinuation within 18 months. Specifically, patients were at increased risk of discontinuation following receipt of residential treatment, chaplain, recreational or occupational therapy, vocational services, criminal justice outreach, PTSD treatment, clinical pharmacy, social work and mental health clinic services (Table 3). SUD psychotherapy was associated with a statistically significant 10% decreased risk of discontinuation, and primary-care-based mental health was associated with a 5% decreased risk of discontinuation.

**Table 3:** Associations between service use and buprenorphine discontinuation within 18 months

Service	Minimally adjusted	Fully adjusted
	HR (95% CI)	HR (95% CI)
Substance use disorder psychotherapy	0.90 (0.86, 0.95)	**0.82 (0.77, 0.87)**
Primary care-based mental health	0.95 (0.90, 1.00)	**0.93 (0.88, 0.99)**
Primary care	0.97 (0.93, 1.01)	**0.94 (0.90, 0.98)**
Pain services	1.00 (0.94, 1.06)	0.96 (0.91, 1.02)
Mental health clinic	1.14 (1.08, 1.20)	1.05 (1.00, 1.11)
Social work	1.14 (1.09, 1.20)	**1.07 (1.03, 1.12)**
Clinical pharmacy	1.15 (1.09, 1.20)	**1.07 (1.02, 1.12)**
Post-traumatic stress disorder treatment	1.19 (1.12, 1.27)	**1.09 (1.02, 1.15)**
Criminal justice outreach	1.26 (1.18, 1.35)	**1.12 (1.04, 1.20)**
Vocational services	1.30 (1.21, 1.40)	**1.09 (1.00, 1.19)**
Recreational or occupational therapy	1.38 (1.29, 1.48)	**1.13 (1.06, 1.21)**
Chaplain	1.39 (1.28, 1.51)	**1.13 (1.05, 1.22)**
Residential treatment	1.50 (1.37, 1.65)	**1.26 (1.15, 1.39)**

Bolding indicates statistically significant fully adjusted HRs after false discovery rate correction. Minimally adjusted models include each lagged service predictor separately, fully adjusted model includes all lagged service predictors simultaneously. All models adjust for age, gender, race, lagged ED visits, and lagged receipt of buprenorphine from multiple facilities. Note. HR = Hazard Ratio. 95% CI = 95% Confidence Interval. ED = emergency department

After holding constant all time-varying and time-stable covariates, a false-discovery-rate corrected statistically significant association remained for residential treatment, which was associated with 26% higher risk of subsequent discontinuation (Table 3). The initial finding of decreased risk for individuals who engaged in SUD psychotherapy also remained: Patients who received SUD psychotherapy were 18% less likely to subsequently discontinue MOUD compared with those who had not engaged in SUD psychotherapy in the fully adjusted model. Although several other services (i.e., chaplain, recreational or occupational therapy, criminal justice outreach, vocational services, PTSD treatment, clinical pharmacy, social work) remained statistically significantly associated with greater risk of discontinuation after false discovery rate correction, these associations were smaller in magnitude (hazard ratios ranging from 1.07 to 1.13). Similarly, primary care and primary-care-based mental health were associated with slightly lower risks of discontinuation (6–7%). The mental health clinic association was attenuated and no longer statistically significant.

### Sensitivity analyses

Overall, we found similar results for the fully adjusted, simultaneous cox regression models across models excluding inpatient/residential quarters to reduce immeasurable time bias, restricted to 6 follow-up months, and varying the outcome definition (Table 4, Table S2, and Table S3, respectively). There were some small differences, such as a 9–10% decrease in risk after pain services receipt in the 30-day discontinuation and 6-month model. Notably, in the model accounting for immeasurable time, SUD psychotherapy was the service most substantially associated with decreased risk of discontinuation (22%), whereas risk of discontinuation after receipt of residential treatment was 47% higher relative to not receiving it (Table 4).

**Table 4:** Associations between service use and buprenorphine discontinuation within 18 months excluding inpatient/residential quarters (N = 11,654)

Service	Fully adjusted
	HR (95% CI)
Substance use disorder psychotherapy	0.78 (0.73, 0.83)
Primary care-based mental health	0.95 (0.89, 1.01)
Primary care	0.97 (0.93, 1.01)
Pain services	1.00 (0.94, 1.06)
Mental health clinic	1.04 (0.98, 1.10)
Social work	1.06 (1.01, 1.11)
Clinical pharmacy	1.07 (1.02, 1.13)
Post-traumatic stress disorder treatment	1.06 (0.99, 1.13)
Criminal justice outreach	1.06 (0.98, 1.15)
Vocational services	1.10 (1.01, 1.20)
Recreational/occupational therapy	1.16 (1.08, 1.24)
Chaplain	1.15 (1.05, 1.26)
Residential treatment	1.47 (1.34, 1.62)

Fully adjusted model includes all lagged service predictors simultaneously and adjusts for age, gender, race, lagged ED visits, and lagged receipt of buprenorphine from multiple facilities. HR = Hazard Ratio. 95% CI = 95% Confidence Interval. ED = emergency department

## Discussion

This study examined buprenorphine discontinuation among a national cohort of Veterans initiating treatment. The high overall discontinuation rate underscores the need for increased efforts to understand factors that support retention in MOUD among patients. Although discontinuation rates were high in all groups, we observed that patients with certain demographic characteristics had higher rates of discontinuation, namely younger patients relative to older age groups, and Black patients relative to other racial/ethnic groups.

Moreover, we identified associations between several services and risk of buprenorphine discontinuation. Only SUD psychotherapy was consistently associated with decreased risk of discontinuation in primary and sensitivity analyses. Risk of discontinuation was 18% relatively lower following SUD psychotherapy compared with not receiving SUD psychotherapy in the primary adjusted model. The slightly lower risk of discontinuation among those who received primary care or primary-care-based mental health services were not observed in all models. This warrants further exploration, as prior research has shown that integration of buprenorphine treatment with primary care is associated with higher treatment retention [44].We also observed modestly greater risk of discontinuation across analyses in quarters following the receipt of chaplain, recreational or occupational therapy, social work, and clinical pharmacy, with residential treatment having the strongest association (26% greater risk in the primary adjusted analysis).

Systematic reviews [28, 30, 31] examining the impact of adjunctive interventions to MOUD have highlighted that the addition of psychosocial interventions has largely not been shown to improve retention. In contrast to these reviews, we found associations between SUD psychotherapy and decreased risk of subsequent discontinuation. This is consistent with results of a recent study that observed that greater prior year involvement in psychotherapy was associated with a decrease in probability of early buprenorphine discontinuation among Veterans [45]. Our results are not intended to be causally determinative given challenges from potential confounding. However, confounding by indication, which occurs when factors involved in selecting patients into a particular treatment (e.g., SUD) also affect the outcome that is being studied (i.e., discontinuation) [46], may in particular be somewhat less of a concern for our SUD psychotherapy finding because all Veterans included in this cohort required treatment for substance use. We also cannot determine which aspects of SUD psychotherapy, if any, may have supported stronger retention in our study. Nevertheless, we note that contingency management, an evidence-based treatment for SUD nationally disseminated in VHA [28, 30, 47], seems to be the exception as an adjunctive therapy that may be particularly beneficial for MOUD retention. Our findings support the continued investigation of this and other adjunctive SUD psychotherapy interventions.

Several services (social work, clinical pharmacy, chaplain, and recreational or occupational therapy) were associated with a small increased risk of buprenorphine discontinuation, and those who engaged with residential treatment had particularly greater risk of later discontinuation compared with those who had not engaged in this service. The finding of greater risk of discontinuation after engaging with these services may simply reflect confounding by indication, wherein those accessing additional services beyond buprenorphine had greater underlying medical, psychological, and social challenges (e.g., more severe OUD, unstable housing, other social determinants of health, grief) and thus greater likelihood of disrupted care. Alternatively, it is also possible that our findings captured the influence of (negative) peer and provider attitudes about the use of MOUD. The observed relationship between residential treatment and discontinuation is particularly concerning and may, for example, reflect an emphasis on abstinence-based programming. To the extent that providers and other residential patients expose patients to medication stigma, or the belief that medication-free abstinence is the only “true” form of recovery, these services could paradoxically decrease retention in MOUD treatment [48, 49]. This possibility is consistent with barriers to implementation of buprenorphine that have been documented in both VHA and other non-VA recovery programs [32, 33, 50]. A study examining OUD related outcomes among individuals treated in a residential or outpatient setting demonstrated that residential treatment was associated with improved overall OUD treatment retention for those not receiving MOUD, but not for those who received MOUD [51].

We also identified patient sociodemographic characteristics associated with treatment retention, using one of the largest studies of factors related to long-term treatment retention among Veterans to date. Similar to prior studies [20, 24, 25, 52], we found that Black individuals had the highest risk of treatment discontinuation relative to individuals in other racial/ethnic groups. There are well-documented barriers to Black patients’ accessing MOUD treatment that could contribute to this disparity [53, 54]. For example, although we did not have access to information on dosage, studies suggest that Black patients may be less likely to receive effective buprenorphine dosage [55], and higher doses of buprenorphine have been associated with improved retention [56]. Additionally, similar to studies of nonveterans [57], the youngest patients in our cohort had the highest rates of buprenorphine discontinuation.

Overall, these results point to several targets for clinical intervention to improve MOUD retention among the most at-risk Veterans, including younger adults, Black patients, and those involved in residential programs. Such interventions could be aimed at reducing medication stigma or views that MOUD is a “crutch” that should not be utilized long-term [58]. This could involve integrating faith-based community initiatives and targeting treatment-positive social support, as experiences with stigma may prevent Black patients from seeking and remaining in treatment [59–61]. Steps could also be taken to address program or provider-level barriers to retention, with a focus on rehabilitative rather than punitive treatment approaches [55]. Rigid program structure and constraints on patients’ lives may be counterproductive for some patients [48, 58, 62]. A recent qualitative analysis identified systemic barriers, including logistical hurdles to getting medications and rule/policy violations, as themes related to buprenorphine discontinuation among patients receiving care within VA Health Care Systems [63]. Finally, if future research supports our finding that at least some SUD psychotherapy may enhance MOUD treatment retention, clinicians involved in the care of Veterans with OUD should facilitate access to evidence-based psychotherapeutic interventions for substance use, such as contingency management [28, 30, 47]. At the same time, many patients benefit from medication alone, so requiring psychosocial treatments in order to receive medication may be a barrier to MOUD treatment and should be avoided [31].

### Limitations

Our findings should be interpreted in light of the limitations of this study. First, although we established temporal order such that services were received before discontinuation, our study was not designed to generate causal inferences given the possibility of residual confounding [25, 64]. Second, we lacked information on the specific therapy modalities and content of the psychosocial and other services and thus are not able to identify which aspects of interventions could have had a meaningful impact on discontinuation. Furthermore, we did not assess other important outcomes that may be significant to patients, such as the impact of psychosocial services on overall health and quality of life [31, 65].

Third, it is possible that patients may have filled but not taken their medications or that they received medications outside of the VHA or in ways that we could not capture in outpatient pharmacy records. It is possible that if Veterans were in the care of a residential program, there could be facility-level differences in how medication supplies are handled at discharge, giving the false impression that these patients discontinued buprenorphine. For example, if a patient was in a residential setting for less than 2 weeks but received a 2-week supply or more of inpatient pharmacy-provided buprenorphine at discharge, this might appear as a discontinuation in outpatient pharmacy records. Similarly, we only assessed VHA service utilization and did not have access to non-VA data on outside MOUD or other service receipt.

Fourth, we examined time to first discontinuation of buprenorphine, an important clinical outcome, but did not assess whether patients re-initiated MOUD after a discontinuation. Future studies could build upon these findings by examining re-entry into MOUD treatment. Fifth, whereas we chose to treat ED visits as a covariate to reduce confounding, it is possible that instability resulting in ED use may also lie on a causal pathway from a given service to discontinuation. Our design could not distinguish between these possibilities, although the mechanism by which visiting the ED would affect risk of discontinuation is unclear. Finally, these results are specific to the Veteran population that utilizes VHA services, which has been shown to have elevated health burden compared with other Veterans [66]. Our results may not be generalizable to services received by other patient populations.

### Conclusions

In this national study of patients receiving VHA services who initiated buprenorphine pharmacotherapy for OUD, more than 4 out of 5 patients experienced a buprenorphine treatment discontinuation within 18 months. Among a wide range of psychosocial and other services, only SUD psychotherapy was consistently associated with lower risk of later treatment discontinuation. In contrast, several services, notably including residential treatment, were associated with greater risk of subsequent discontinuation, suggesting a need for interventions to improve retention among this high-risk group. Overall, these findings emphasize the need for future studies to explore beneficial and disruptive components of psychosocial services to inform interventions to support MOUD retention among Veterans.

## Supplementary Information

Supplementary Material 1

## Data Availability

These data must remain on Department of Veterans Affairs servers. Investigators interested in using these data for analyses should email the corresponding author.
