# Supplementary material for: Buprenorphine discontinuation and utilization of psychosocial services: a national study in the Veterans Health Administration
Source: Addict Sci Clin Pract. 2025 Apr 16;20:35. doi: 10.1186/s13722-025-00562-1 (PMC12004863; doi:10.1186/s13722-025-00562-1)
Supplement: Supplementary file 1 — Supplementary Material 1 [file 13722_2025_562_MOESM1_ESM.docx]

**Figure S1. Exclusions flow diagram to create study population for primary analysis**


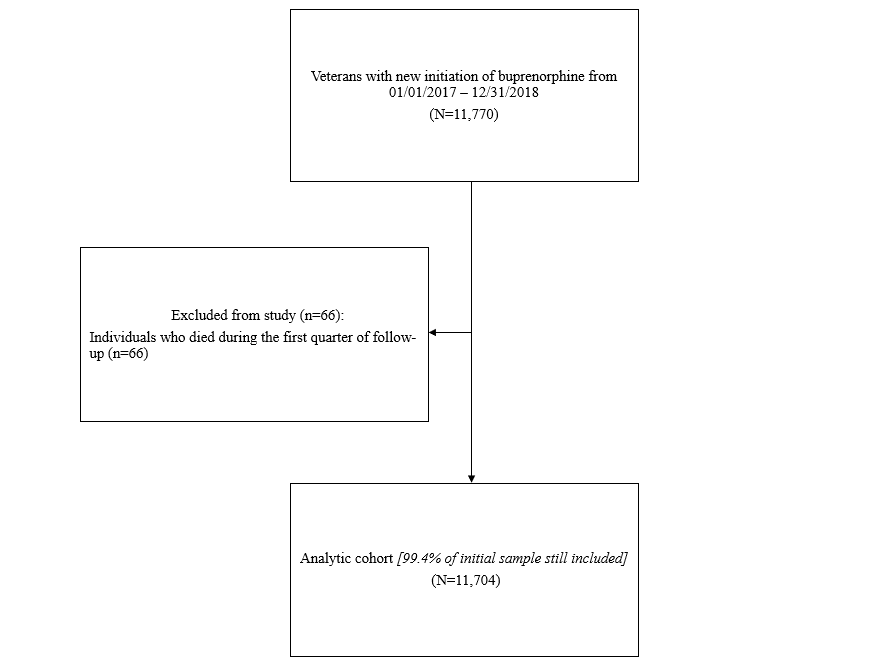


**Table S1 Unadjusted associations between service use and buprenorphine discontinuation in primary analysis**

| **Service** | **N (%) Individuals with service** | **Discontinuations** | **PQs** | **Discontinuation  rate/100 PQs** |
| --- | --- | --- | --- | --- |
| Social work |  |  |  |  |
| None | -- | 3891 | 15028 | 25.9 |
| Any | 8125 (69.4) | 5455 | 14948 | 36.5 |
| Clinical pharmacy |  |  |  |  |
| None | -- | 5700 | 19872 | 28.7 |
| Any | 6139 (52.5) | 3646 | 10104 | 36.1 |
| Recreational or occupational therapy |  |  |  |  |
| None | -- | 7404 | 26257 | 28.2 |
| Any | 3077 (26.3) | 1942 | 3719 | 52.2 |
| Chaplain |  |  |  |  |
| None | -- | 8188 | 27830 | 29.4 |
| Any | 1827 (15.6) | 1158 | 2146 | 54.0 |
| Primary care |  |  |  |  |
| None | -- | 2681 | 9185 | 29.2 |
| Any | 9679 (82.7) | 6665 | 20791 | 32.1 |
| Primary care-based mental health |  |  |  |  |
| None | -- | 8319 | 27292 | 30.5 |
| Any | 2162 (18.5) | 1027 | 2684 | 38.3 |
| Pain services |  |  |  |  |
| None | -- | 8244 | 26554 | 31.0 |
| Any | 1876 (16.0) | 1102 | 3422 | 32.2 |
| Mental health clinic |  |  |  |  |
| None | -- | 2260 | 8824 | 25.6 |
| Any | 9742 (83.2) | 7086 | 21152 | 33.5 |
| Substance use disorder psychotherapy |  |  |  |  |
| None | -- | 2957 | 8268 | 35.8 |
| Any | 8755 (74.8) | 6389 | 21708 | 29.4 |
| Post-traumatic stress disorder treatment |  |  |  |  |
| None | -- | 8495 | 27848 | 30.5 |
| Any | 1447 (12.4) | 851 | 2128 | 40.0 |
| Vocational services |  |  |  |  |
| None | -- | 8592 | 28192 | 30.5 |
| Any | 1280 (10.9) | 754 | 1784 | 42.3 |
| Criminal justice outreach |  |  |  |  |
| None | -- | 8657 | 28398 | 30.5 |
| Any | 1037 (8.9) | 689 | 1578 | 43.7 |
| Residential treatment |  |  |  |  |
| None | -- | 7879 | 27405 | 28.8 |
| Any | 2017 (17.2) | 1467 | 2571 | 57.1 |
| Emergency department |  |  |  |  |
| None | -- | 6116 | 22723 | 26.9 |
| Any | 5326 (45.5) | 3230 | 7253 | 44.5 |
| Buprenorphine from multiple facilities |  |  |  |  |
| None | -- | 9268 | 29673 | 31.2 |
| Any | 283 (2.4) | 78 | 303 | 25.7 |

*Note.* Services were lagged by one quarter so that individuals were considered exposed in each quarter if they were involved in the service in the prior quarter. Individuals with service refers to lagged service receipt for quarters that contributed to analysis only. PQs=person quarters.

**Table S2 Associations between service use and buprenorphine discontinuation within 6 months**

| **Service** | **Fully Adjusted** |
| --- | --- |
|  | **HR (95% CI)** |
| Substance use disorder psychotherapy​ | 0.82​ (0.77, 0.87) |
| Primary care-based mental health | 0.91​ (0.85, 0.96) |
| Primary care​ | 0.92 (0.88, 0.96)​ |
| Pain services​ | 0.90​ (0.84, 0.97)​ |
| Mental health clinic​ | 1.04​ (0.98, 1.10)​ |
| Social work​ | 1.08 (1.03, 1.13)​ |
| Clinical pharmacy​ | 1.06​ (1.01, 1.11)​ |
| Post-traumatic stress disorder treatment​ | 1.08​ (1.02, 1.16)​ |
| Criminal justice outreach​ | 1.09 (1.00, 1.19)​ |
| Vocational services​ | 1.09​ (1.00, 1.19)​ |
| Recreational therapy/occupational therapy​ | 1.13​ (1.06, 1.20)​ |
| Chaplain​ | 1.12​ (1.03, 1.21)​ |
| Residential treatment | 1.27​ (1.16, 1.40​) |

*Note.* Fully adjusted model includes all lagged service predictors simultaneously and adjusts for age, gender, race, lagged emergency room visits, and lagged receipt of buprenorphine from multiple facilities. HR= Hazard Ratio. 95 % CI= 95% Confidence Interval.

**Table S3 Associations between service use and buprenorphine discontinuation within 18 months using alternative 30-day discontinuation definition**

| **Service** | **Fully Adjusted** |
| --- | --- |
|  | **HR (95% CI)** |
| Substance use disorder psychotherapy​ | 0.85​ (0.80, 0.90) |
| Primary care-based mental health | 0.88​ (0.82, 0.95) |
| Primary care​ | 0.95 (0.91, 0.99)​ |
| Pain services​ | 0.91​ (0.85, 0.98)​ |
| Mental health clinic​ | 1.07​ (1.02,1.13)​ |
| Social work​ | 1.11 (1.06, 1.16)​ |
| Clinical pharmacy​ | 1.06​ (1.01, 1.11)​ |
| Post-traumatic stress disorder treatment​ | 1.06 (0.99, 1.13)​ |
| Criminal justice outreach​ | 1.11 (1.03, 1.19)​ |
| Vocational services​ | 1.08​ (0.99, 1.19)​ |
| Recreational therapy/occupational therapy​ | 1.17​ (1.09, 1.24)​ |
| Chaplain​ | 1.14​ (1.04, 1.24)​ |
| Residential treatment | 1.25​ (1.12, 1.39​) |

*Note.* Fully adjusted model includes all lagged service predictors simultaneously and adjusts for age, gender, race, lagged emergency room visits, and lagged receipt of buprenorphine from multiple facilities. HR= Hazard Ratio. 95 % CI= 95% Confidence Interval.
